# Discovery and replication of blood-based proteomic signature of PTSD in 9/11 responders

**DOI:** 10.1038/s41398-022-02302-4

**Published:** 2023-01-11

**Authors:** Monika A. Waszczuk, Pei-Fen Kuan, Xiaohua Yang, Jiaju Miao, Roman Kotov, Benjamin J. Luft

**Affiliations:** 1 Department of Psychology, Rosalind Franklin University of Medicine and Science, North Chicago, IL USA; 2 Department of Applied Mathematics and Statistics, Stony Brook University, Stony Brook, NY USA; 3 Department of Medicine, Stony Brook University, Stony Brook, NY USA; 4 Department of Psychiatry, Stony Brook University, Stony Brook, NY USA

**Keywords:** Predictive markers, Psychiatric disorders

## Abstract

Proteomics provides an opportunity to develop biomarkers for the early detection and monitoring of post-traumatic stress disorder (PTSD). However, research to date has been limited by small sample sizes and a lack of replication. This study performed Olink Proseek Multiplex Platform profiling of 81 proteins involved in neurological processes in 936 responders to the 9/11 disaster (mean age at blood draw = 55.41 years (SD = 7.93), 94.1% white, all men). Bivariate correlations and elastic net regressions were used in a discovery subsample to identify concurrent associations between PTSD symptom severity and the profiled proteins, and to create a multiprotein composite score. In hold-out subsamples, nine bivariate associations between PTSD symptoms and differentially expressed proteins were replicated: SKR3, NCAN, BCAN, MSR1, PVR, TNFRSF21, DRAXIN, CLM6, and SCARB2 (|*r*| = 0.08–0.17, *p* < 0.05). There were three replicated bivariate associations between lifetime PTSD diagnosis and differentially expressed proteins: SKR3, SIGLEC, and CPM (*OR* = 1.38–1.50, *p* < 0.05). The multiprotein composite score retained 38 proteins, including 10/11 proteins that replicated in bivariate tests. The composite score was significantly associated with PTSD symptom severity (*β* = 0.27, *p* < 0.001) and PTSD diagnosis (*OR* = 1.60, 95% CI: 1.17–2.19, *p* = 0.003) in the hold-out subsample. Overall, these findings suggest that PTSD is characterized by altered expression of several proteins implicated in neurological processes. Replicated associations with TNFRSF21, CLM6, and PVR support the neuroinflammatory signature of PTSD. The multiprotein composite score substantially increased associations with PTSD symptom severity over individual proteins. If generalizable to other populations, the current findings may inform the development of PTSD biomarkers.

## Introduction

Post-traumatic stress disorder (PTSD) is a persistent, debilitating psychiatric condition [1, 2] affecting up to 30% of people in high-risk groups, such as veterans and first responders [3–6]. PTSD can have deleterious physical consequences that place substantial strain on both individuals and the healthcare system [7]. Biological alterations associated with PTSD might provide an opportunity to improve detection and intervention for this condition.

Because PTSD has a complex etiology, a downstream biological signature should capture both genetic and environmental contributions to PTSD, and should be functionally proximal to the clinical presentation. Proteins meet this requirement because they perform most functions within cells and facilitate intercellular communication. Protein expression can reflect an organism’s biological state, including disease-related alterations that are not detectable at the genetic or transcript level. Thus, proteins might plausibly play roles in pathological processes underpinning PTSD and serve as PTSD biomarkers.

Although a single candidate protein is unlikely to capture the complex pathophysiology of PTSD, a panel of proteins might more accurately reflect PTSD disease status. Recent progress in clinical proteomic technologies has enabled information on protein expression to be collected from many proteins in peripheral tissues, such as the plasma. Therefore, proteomics is a preferred approach for unbiased biomarker discovery.

To date, proteomics has advanced the understanding of several psychiatric disorders, including schizophrenia, bipolar disorder, and depression [8–10]. For example, proteomics profiles derived from machine learning approaches have been found to discriminate between bipolar disorder and major depressive disorder in several independent studies, with a range from AUC = 0.67 [11] to AUC = 0.81 [12].

Differential expression of peripheral proteins has been reported in PTSD, most consistently for inflammatory cytokines [13, 14]. To our knowledge, the largest proteomics study in PTSD, exploring the expression profiles of 276 proteins, was recently conducted by our team [15]. In that study, machine learning identified 38 differentially expressed proteins in World Trade Center (WTC) responders with a probable PTSD diagnosis. Multiprotein composite scores based on the top differentially expressed proteins achieved a high accuracy in the classification of cases vs controls (AUC = 0.77–0.83). Eighteen of the top differentially expressed proteins were predominantly involved in a range of neurobiological and neuroinflammatory processes. This is in line with the well-established evidence that genes implicated in PTSD are expressed in several cortical and subcortical regions [16–19], as well as with findings of widespread immune dysregulation in PTSD [20]. These initially promising results must be replicated in independent samples to better validate their biomarker potential.

Overall, a growing body of work has demonstrated that incorporating proteomic profiling strategies might elucidate the pathophysiology of psychiatric conditions and provide a biological basis for detection and intervention. It is important to note that processes such as diurnal variation in protein expression, interactions with other cells in the blood, and patient-related factors such as diet can affect peripheral measurements of proteomics. Nonetheless, plasma remains a more accessible tissue than the brain or specific cells for clinical translation purposes. The existing blood-based proteomics literature is further limited in several aspects [21]. Notably, sample sizes in prior studies have generally been small and have been further subdivided into discovery and test sets for machine learning purposes. Furthermore, replication across studies is lacking. Finally, most studies have used a case-control design, which lowers statistical power and is vulnerable to confounds such as case-control differences in socio-economic status, medical comorbidities, and treatment exposure associated with treatment-seeking [22, 23]. In line with ample evidence that biological vulnerability transcends diagnostic boundaries [24], proteomics might benefit from dimensional psychiatric definitions.

To address these limitations and to build on previous findings from Kuan et al. [15], the current study investigated associations between proteomics and PTSD in a much larger, independent sample of WTC responders. A representative cohort of more than 900 responders with a full range of PTSD symptoms and lifetime PTSD diagnostic data were profiled for 92 proteins involved in neurobiological and neuroinflammatory processes. First, we attempted to replicate findings for the 18 neurology panel proteins reported to be differentially expressed in Kuan et al. [15]. Second, to test for associations between PTSD and all profiled proteins in the current sample, we divided the sample into discovery and hold-out (replication) subsamples. In the discovery subsample, we established bivariate associations and derived a multiprotein composite score for dimensional PTSD symptom severity. The discovered associations were then replicated against PTSD symptoms as well as clinical PTSD diagnosis in the hold-out subsample.

## Methods

### Participants

The participants were 1000 male WTC responders enrolled in the Stony Brook University WTC Health Program [25]. This program monitors more than 10,000 WTC responders, primarily from Long Island, NY. Blood samples were drawn routinely during monitoring examinations at the WTC Health Program. Only male participants with available blood samples were randomly selected for participation in the project. We only assayed samples from male responders because women make up <10% of the cohort and show protein expression patterns notably different from those in men [26]. The study was approved by the Institutional Reviewer Board of Stony Brook University, and all participants provided written informed consent to participate.

Sixteen samples that failed QC and 48 duplicate IDs were excluded, thus resulting in an analytic *N* = 936. The mean age at blood draw was 55.41 years (SD = 7.93, range = 37–81 years); 94.1% of participants were white, 94.2% were non-Hispanic, 64.4% were employed in law enforcement on 9/11, and 19.3% were exposed to a toxic dust cloud during 9/11. Full demographic and clinical information about the study sample is reported in Supplementary Table 1. The assayed sample is representative of the 9/11 responder population at the Health Program. None of the participants were previously included in the proteomics study by Kuan et al. [15].

### PTSD measures

PTSD symptoms were assessed with the PTSD Checklist (PCL)–Specific Version [27]. PCL-17 is a 17-item self-reported questionnaire assessing the severity of WTC-related DSM-IV PTSD symptoms in the past month, on a five-point scale (1 = not at all to 5 = extremely). PCL-17 has been demonstrated to have sound psychometric properties [28] and had excellent internal consistency in the current sample (α = 0.96). The PTSD symptom assessment was concurrent with the blood draws in 70.8% of the sample; otherwise, the most recent available PTSD assessment was used. The mean lag between PTSD symptom assessment and the blood draw in the full sample was 14.59 days (SD = 26.85, range = 0–196 days), and among participants without a concurrent blood draw, it was 54.33 days (SD = 22.96, range = 1–196 days).

Two sources of diagnostic interview data were available to obtain the lifetime PTSD diagnosis. First, master’s level clinical assessors were trained to administer selected modules of the Structured Clinical Interview for DSM-IV [29] for a study of WTC PTSD [6]. The second source of diagnostic data came from the Diagnostic Interview Schedule for DSM-IV [30], which was administered by trained mental health professionals to every responder at the second monitoring visit and at follow-up visits if necessary [25, 31]. Lifetime diagnosis was ascertained if responders met the diagnostic criteria at least once, according to either the Structured Clinical Interview for DSM-IV or the Diagnostic Interview Schedule for DSM-IV. Both interviews were modified to assess PTSD symptoms associated with traumatic WTC exposures (criterion A). Participants reported information on their worst episode since 9/11. The inter-rater agreement for 55 independently rated audio tapes was very good (κ ≥ 0.82) [6]. Overall, diagnostic data were available on *N* = 787, 84.1% of the total sample, resulting in 122 lifetime WTC-related PTSD cases (15.5% prevalence rate). PTSD cases and controls differed significantly on PTSD symptom severity (*t*(*df*) = 16.87 (768), *p* < 0.001, *d* = 11.62) and prevalence of lifetime depression diagnosis (*X*^2^(1, 766) = 184.92, *p* < 0.001, *φ* = 0.49), but not on any demographic and other clinical characteristics, see Supplementary Table 1. No significant differences were observed in demographic characteristics and PCL severity between responders with or without diagnostic interview data.

### Proteomics profiling

Fasting plasma samples were collected from the morning to noon during the participant’s monitoring visit. Blood was collected in two BD Vacutainer blood collection tubes with K2EDTA and centrifuged (2000×*g*, 4 °C for 10 min). Plasma samples were aliquoted into a 1.5 ml polypropylene vial (0.5 ml plasma in each vial) and stored at a −80 °C freezer within 30 min of blood collection. All plasma samples were kept at −80 °C freezer until analysis. Plasma protein expression was profiled with the Olink Proseek Multiplex Platform. The Olink multiplex immunoassay was designed to provide an ultrasensitive, reproducible, highly multiplexed method for measuring protein expression. The measurement was based on state-of-the-art proximity extension assay technology [32]. We used the Olink Neurology panel consisting of 92 proteins, including markers associated with neurobiological processes and neurological diseases (e.g., neural development, axon guidance, synaptic function, or specific conditions such as Alzheimer’s disease), as well as with broader roles in processes such as cellular regulation, immunology, development, and metabolism. More details are available from the manufacturer online (https://www.olink.com).

### Proteomics data preprocessing

Several internal and external controls were added to the plasma samples for quality control to monitor protein–antibody reactions, the DNA extension step, and the detection quality of the qPCR, in order to estimate the background signal and calculate the limit of detection for Olink panels. All values below the limit of detection were coded as missing. A total of 11 proteins were excluded from the panel because of a high missing rate or failed QC, thus resulting in a final panel of 81 proteins. Proteomics data are presented as normalized protein expression values, Olink Proteomics’ arbitrary units on a log scale, i.e., a difference of one normalized protein expression unit indicated a doubling of protein concentration.

### Analytic approach

First, we tested whether the 18 proteins from the Olink Neurology panel that were differentially expressed in the PTSD groups in Kuan et al. [15] were significantly associated with PTSD symptom severity and PTSD diagnosis, in the full sample. Associations were adjusted for age and the time lag between PTSD assessment and blood draw, and a 5% FDR correction was applied.

Second, the total sample was randomly split in a 7:3 ratio into discovery (*N* = 657) and hold-out (*N* = 279) subsamples. Given that diagnostic data were used only for test purposes, the random split was conditional on PTSD diagnostic information being available for participants in the hold-out subsample. The discovery and hold-out subsamples did not significantly differ in PTSD diagnosis prevalence (*X*^2^(1, *N* = 787) = 1.93, *p* = 0.17, *φ* = −0.05) and PCL severity (*t*(482.45) = 1.49, *p* = 0.14, *d* = 0.11). All analyses were adjusted for age and the time lag between PTSD assessment and blood draw.

Partial correlations between PCL symptom severity and the expression levels of the remaining 63 proteins (i.e., excluding significant proteins from Kuan et al. analyzed above) were calculated in the discovery subsample. Proteins with associations at nominal *p* < 0.05 in the discovery subsample were taken forward to the hold-out subsample. Partial correlations between proteins and PCL symptom severity, and logistic regressions between proteins and lifetime PTSD diagnosis, in the hold-out subsample, were considered statistically significant replications at nominal *p* < 0.05.

To jointly test the association between PTSD and all relevant proteins, we created a multiprotein composite score by using elastic net regression. All 81 proteins and covariates were used as a candidate feature set in the discovery subsample. The optimal tuning parameters were determined via fivefold cross-validation. Next, in the hold-out subsample, linear and logistic regressions were used to estimate the associations between the multiprotein composite scores and the PCL symptom severity and PTSD diagnosis, respectively.

## Results

Of the 18 neurology panel proteins that emerged in Kuan et al. [15], seven showed significant bivariate associations with PTSD symptom severity at the 5% FDR level: BCAN, NCAN, MSR1, PVR, TNFRSF21, SKR3, and DRAXIN (|*r*| = 0.08–0.14; Table 1). Only SKR3 was associated with PTSD diagnosis at the 5% FDR level (*OR* = 1.38, 95% CI: 1.14–1.68). Nearly all (16/18) proteins showed a sign consistent with that in Kuan et al. [15] across PTSD symptoms and PTSD diagnosis comparisons (*P* for a sign test for consistency = 7.0 × 10^−4^).

**Table 1:** Bivariate associations between proteins discovered in Kuan et al. (2020) and PTSD symptoms and diagnosis in the current sample.

Kuan et al., 2020		Total sample			
Significant proteins	Direction	PTSD symptoms (*N* = 936)		PTSD Diagnosis (*N* = 787)	
		*r*	*p* value	OR (95% CI)	*p* value
**SKR3**	**Up**	**0.14**	**0.000**	**1.38 (1.14–1.68)**	**0.001**
**NCAN**	**Down**	**−0.12**	**0.001**	0.80 (0.66-0.97)	0.022
**BCAN**	**Down**	**−0.10**	**0.001**	0.84 (0.68–1.02)	0.075
**MSR1**	**Up**	**0.11**	**0.001**	1.28 (1.04–1.58)	0.018
**PVR**	**Up**	**0.09**	**0.006**	1.24 (1.02–1.52)	0.035
**TNFRSF21**	**Up**	**0.10**	**0.002**	1.27 (1.03–1.56)	0.023
**DRAXIN**	**Up**	**0.08**	**0.018**	1.14 (0.94–1.38)	0.193
CTSS	Up	0.05	0.139	1.12 (0.92–1.36)	0.256
MDGA1	Up	0.04	0.201	1.23 (1.01–1.51)	0.041
CPA2	Up	0.01	0.713	1.12 (0.91–1.36)	0.283
EDA2R	Up	0.03	0.447	1.27 (1.00–1.62)	0.052
FLRT2	Up	0.04	0.237	1.30 (1.06–1.60)	0.011
ADAM22	Down	−0.05	0.130	1.03 (0.84–1.26)	0.775
CTSC	Up	0.00	0.915	1.12 (0.93–1.36)	0.227
CD200	Up	0.03	0.357	1.29 (1.06–1.58)	0.013
DDR1	Up	0.05	0.160	1.11 (0.91–1.35)	0.325
sFRP3	Up	−0.01	0.666	1.06 (0.87–1.29)	0.537
VWC2	Up	0.05	0.125	1.28 (1.04–1.58)	0.022

Replicated results after 5% FDR correction are bolded. Associations were adjusted for age and the time lag between PTSD assessment and blood draw.

Across the remaining 63 profiled proteins in the current study, nine showed significant bivariate associations with PTSD symptom severity in the discovery subsample (Table 2). In the hold-out subsample, we replicated two associations with PTSD symptom severity: SCARB2 (*r* = 0.17) and CLM6 (*r* = 0.13), as well as replicated two associations with PTSD diagnosis: CPM (*OR* = 1.50, 95% CI: 1.08–2.09) and SIGLEC1 (*OR* = 1.45 CI: 1.04–2.03). Across the discovery and hold-out subsamples, all nine proteins were associated in a consistent direction with PTSD symptom severity, and 7/9 were associated in a consistent direction with PTSD diagnosis. Overall, 11 unique protein markers were associated with PTSD; their functional pathways included cell adhesion, cellular metabolic processes, neurogenesis, and immune response (Table 3).

**Table 2:** Bivariate associations between significant proteins in the discovery subsample, and PTSD symptoms and diagnoses in the hold-out subsample.

Discovery subsample (*N* = 657)		Hold-out subsample (*N* = 279)			
Significant proteins	Direction	PTSD symptoms		PTSD diagnosis	
		*r*	*p* value	OR (95% CI)	*p* value
**CLM6**	**Up**	**0.13**	**0.031**	1.28 (0.94–1.75)	0.116
**SCARB2**	**Up**	**0.17**	**0.006**	1.36 (0.99–1.86)	0.057
**SIGLEC1**	**Up**	0.10	0.091	**1.45 (1.04–2.03)**	**0.030**
**CPM**	**Up**	0.10	0.094	**1.50 (1.08–2.09)**	**0.016**
RGMA	Down	−0.07	0.263	0.99 (0.73–1.36)	0.969
SMPD1	Up	0.10	0.120	0.96 (0.70–1.31)	0.786
CNTN5	Down	−0.10	0.088	0.77 (0.55–1.07)	0.114
CLM1	Up	0.09	0.132	1.20 (0.85–1.70)	0.307
TNR	Down	–0.03	0.592	1.10 (0.80–1.50)	0.560

Replicated results at nominal *p* < 0.050 are bolded. Associations were adjusted for age and the time lag between PTSD assessment and blood draw.

**Table 3:** Functional pathways of 11 replicated proteins associated with PTSD.

Replicated protein	PTSD definition	Functional pathways
SKR3	Symptom severity and diagnosis	Cell adhesion, cell differentiation, cell growth, cellular metabolic process, signal transduction
NCAN	Symptom severity	Cell adhesion, Cellular metabolic process
BCAN	Symptom severity	Cell adhesion, Cellular metabolic process
MSR1	Symptom severity	Cell differentiation
PVR	Symptom severity	Cell adhesion, immune response
TNFRSF21	Symptom severity	Cell adhesion, cell death, cell differentiation, immune response, neurogenesis, signal transduction
DRAXIN	Symptom severity	Axon development, axon guidance, cell death, cell differentiation, cell growth, neurogenesis, signal transduction
CLM6	Symptom severity	Immune response
SCARB2	Symptom severity	Membrane transportation
CPM	Diagnosis	Cellular metabolic process, Proteolysis
SIGLEC1	Diagnosis	Cell adhesion

**Fig. 1:**
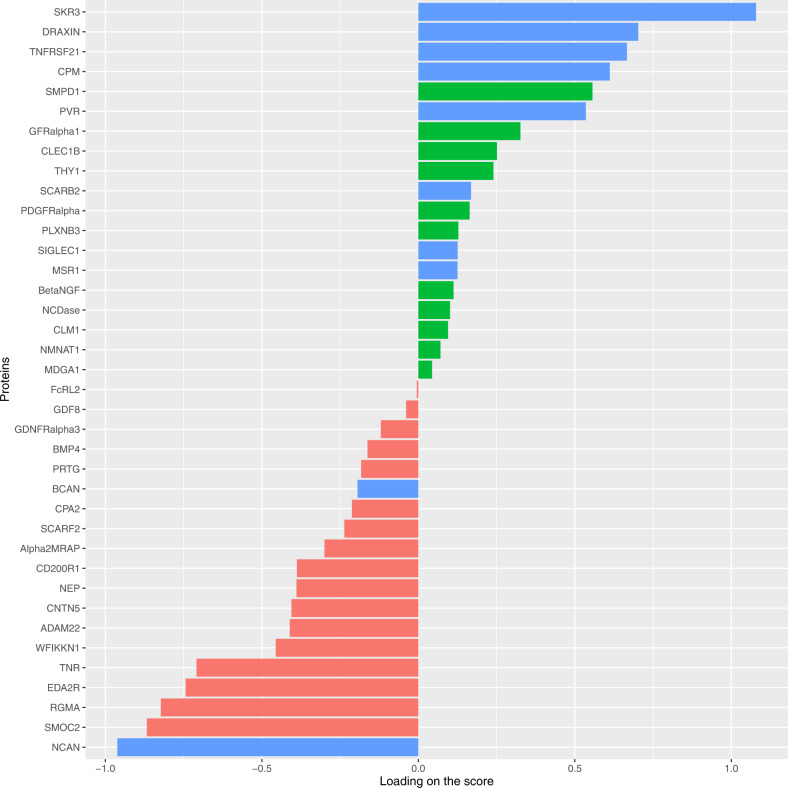
Proteins retained in the multiprotein composite score. Replicated proteins from bivariate analyses of PTSD symptom severity and PTSD diagnosis are denoted by the blue bars.

**Fig. 2:**
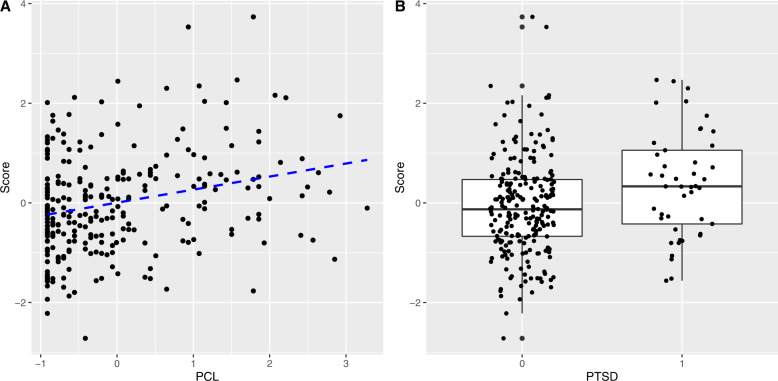
The associations between the multiprotein score and A PTSD symptoms and B PTSD diagnosis, in the hold-out subsample (*N* = 279). Associations between the multiprotein score and PTSD were adjusted for age and the time lag between PTSD assessment and blood draw.

The multiprotein composite score created in the discovery subsample retained 38 differentially expressed proteins (Fig. 1). The proteins that loaded most strongly on the composite score were NCAN and SKR3. The composite score retained 10/11 differentially expressed proteins that replicated in bivariate tests, all with a consistent direction of effect. Finally, in the hold-out subsample, the multiprotein score was significantly associated with PCL severity (*β* = 0.27, *p* < 0.001) and with PTSD diagnosis status (*OR* = 1.60, 95% CI: 1.17–2.19, *p* = 0.003) (Fig. 2). Sensitivity analyses demonstrated that additional demographic covariates—race, Hispanic ethnicity, employment in law enforcement during 9/11, and 9/11 dust cloud exposure—did not change the findings.

## Discussion

To our knowledge, the current study reports the largest discovery and replication of blood-based proteomics findings in PTSD to date. By analyzing dimensional PTSD symptom severity in over 900 responders to the 9/11 disaster, we replicated findings for seven proteins reported previously in an independent study, identified and replicated two new protein markers associated with PTSD symptom severity, and two associated with PTSD diagnosis. Overall, we report a total of 11 unique replicated proteins associated with PTSD. Finally, we derived the multiprotein composite score and demonstrated that it significantly predicted PTSD symptom severity, as well as diagnostic status, in a hold-out sample. Together, the current findings make an important contribution to the understanding of the pattern of differential protein expression characterizing PTSD. If generalizable to other populations, they may aid in the development of biomarkers for detecting and monitoring PTSD.

The current study replicated findings for seven differentially expressed proteins that were originally discovered by Kuan et al. (2020). Most notably, serine/threonine-protein kinase receptor R3 (SKR3) was associated with both dimensional PTSD symptom severity and PTSD diagnosis in the current sample. SKR3 is expressed in neurons and regulates normal blood vessel development.

The remaining six proteins replicated only in association with dimensional PTSD symptom severity. Neurocan (NCAN) and brevican (BCAN) were found to be downregulated in patients with higher PTSD symptom severity. NCAN is thought to be involved in modulating cell adhesion and migration, whereas BCAN plays a role in the formation of the brain’s extracellular matrix, and has been implicated in brain development and synaptic plasticity. The NCAN gene variants independently emerged as genome-wide-significant risk loci for bipolar disorder and schizophrenia [33], as well as a major depressive disorder [34]. Similarly, both the NCAN and BCAN genes have been found to be differentially expressed in postmortem brains of patients with schizophrenia [35], and differential NCAN gene expression has been reported in PTSD in a blood-based sample [36], but NCAN and BCAN expression was non-significant in postmortem brain samples of PTSD patients [17, 19].

The third replicated protein, macrophage scavenger receptor types I and II (MSR1, also known as CD204), has been implicated in many macrophage-associated physiological and pathological processes, including atherosclerosis, Alzheimer’s disease, prostate cancer, and host defense [37]. The MSR1 gene has also been found to be differentially expressed in PTSD [38]. The fourth replicated protein, poliovirus receptor (PVR), has multiple roles in the immune response, including mediating NK cell adhesion and triggering their effector functions. PVR has been found to be associated with bipolar disorder, schizophrenia [39], and Alzheimer’s disease [40, 41].

The fifth replicated protein, tumor necrosis factor receptor superfamily member 21 (TNFRSF21), plays a role in neuronal apoptosis and the negative regulation of oligodendrocyte maturation [42]. The TNFRSF21 gene has been found to be differentially expressed in PTSD [43, 44], including in postmortem brain samples in orbitofrontal cortex and dorsal anterior cingulate cortex regions [17], and genetic variants within this gene have emerged in genome-wide association studies of panic disorder [45] and depression [46, 47]. Finally, DRAXIN plays a role in neural development and has been implicated in autism spectrum disorder and obsessive-compulsive disorder [48].

The current study discovered and replicated four additional proteins associated with PTSD. Two proteins were upregulated in patients with higher PTSD symptom severity. The first, CMRF35-like molecule 6 (CLM6, also known as CD300c), is an activating receptor expressed on monocytes that play a role in immune system processes [49]. The second, lysosome membrane protein 2 (SCARB2, also known as CD36), is a glycoprotein that is located in the membranes of lysosomes and endosomes, and may participate in membrane transportation [50]. Differential gene expression of SCARB2 has been found to be significantly associated with PTSD [36], and genetic variants within the SCARB2 gene have been associated with Parkinson’s disease in several genetic studies [51, 52]. Of note, although CLM6 and SCARB2 are intracellular proteins, they can be released into plasma during natural cell processes as well as sample storage [53].

Two proteins found in the discovery subsample were significantly associated with PTSD diagnosis in the hold-out subsample. The first, sialoadhesin (SIGLEC1), is a cell adhesion molecule found on the surfaces of macrophages. Differential gene expression of SIGLEC1 has been found to be significantly associated with PTSD [36]. The second, carboxypeptidase M (CPM), is an enzyme that is associated with monocyte-to-macrophage differentiation and has been implicated in metabolizing bioactive peptides, hormones, and cytokines [54].

To investigate the clinical utility of our proteomic findings, we constructed a PTSD composite multiprotein score by aggregating 38 proteins selected in the discovery sample using machine learning. All replicated proteins discussed above, with the exception of CLM6, contributed to the score, together with proteins that did not reach significance in bivariate comparisons. The composite multiprotein score significantly predicted PTSD symptom severity and diagnostic status in the independent hold-out sample, suggesting that the score indicates illness activity and severity, and may potentially aid in treatment monitoring. Although the current findings are promising, future longitudinal studies are needed to establish whether the multiprotein score might predict the onset and/or chronicity of future PTSD after trauma exposure.

### Limitations

The strengths of the current study include its use of a state-of-the-art multiplex proteomics approach to profile a comprehensive and validated panel of neurology proteins; the replication of results in independent subsamples; and the inclusion of many participants, all of whom were exposed to a common trauma. Nonetheless, this study has several notable limitations. First, because the reported associations are cross-sectional, we were unable to determine whether the observed alterations in protein expression were a consequence of PTSD or part of its etiology. The inclusion of trauma-exposed participants without PTSD symptoms suggested that the proteomic signature is not just a consequence of trauma but is linked to PTSD; however, a longitudinal study design is needed to determine the direction of the association of protein expression with PTSD. Second, the current results must be replicated in women to better understand sex differences in protein expression, and in more diverse cohorts with other trauma exposures to clarify the generalizability of the findings.

Third, although blood testing is a relatively feasible, scalable, and non-invasive approach for obtaining biomarkers and could serve as a first step of the multistage diagnostic process, there are notable considerations for relying on blood-based proteomics in neuro-psychiatric conditions [55–58]. Protein expression in the blood reveals the proteins secreted into the blood from multiple organs, tissues, and cells. All proteins reported here are involved in neurobiology and the brain is their most likely source, but this needs to be verified in postmortem studies. Moreover, proteins originating from the brain and the central nervous system might be present at low concentrations in blood once they have crossed the blood–brain barrier, with the dilution confounded by interactions with other plasma proteins and blood cells, as well as by degradation processes in the blood and in the liver. Diurnal variations in protein concentrations can further confound the findings, given that blood was drawn at various times throughout the morning in the current study. Numerous other factors can influence the measurement of protein expression, including demographic characteristics, medication use, diet, and other lifestyle factors. Although fasting plasma samples were collected, in the current large population-based study, we were not able to control all of these factors. However, the confounds likely have contrasting effects on protein expression, and while potentially lowering the overall observed associations, they reflect the clinical reality of translating proteomic findings to clinical practice. Studies of cognitive performance, Alzheimer’s disease, and others demonstrate the utility and validity of studying neurology-related proteins in blood [58–60]. Finally, whole blood is a complex tissue and protein expression could differ if the study used serum instead of plasma, or utilized a different blood processing or storage pipeline. The current study also was not designed to test cell-specific protein expression.

## Conclusions

The current study reports replication of findings for 11 differentially expressed proteins associated with PTSD, many of which play active roles in neuroinflammatory mechanisms and have been implicated in psychiatric and neurologic conditions in prior studies. The multiprotein composite score enabled significant prediction of dimensional PTSD symptom severity as well as PTSD diagnosis in a hold-out sample. Therefore, proteomics might be applied to develop useful biomarkers of PTSD for research and clinical practice. The current results will require replication in other traumatized populations to better understand their generalizability.

## Supplementary information

Supplementary Materials

## Data Availability

The proteomics data will be available at synapse.org upon publication (https://www.synapse.org/#!Synapse:syn32140679, 10.7303/syn32140679).
