# Supplementary material for: Discovery and replication of blood-based proteomic signature of PTSD in 9/11 responders
Source: Transl Psychiatry. 2023 Jan 11;13:8. doi: 10.1038/s41398-022-02302-4 (PMC9834302; doi:10.1038/s41398-022-02302-4)
Supplement: Supplementary file 1 — Supplementary Materials [file 41398_2022_2302_MOESM1_ESM.docx]

**SUPPLEMENTARY MATERIALS**

Supplementary Table 1 – Demographic and clinical characteristics of the study sample

|  | **Total sample (N=936)** | **Diagnosed subsample (N=787)** | |
| --- | --- | --- | --- |
|  |  | **PTSD cases (N=122)** | **PTSD controls (N=665)** |
| **Mean (SD, range)** |  |  |  |
| Age at blood draw | 55.41 (7.93, 37–81) | 55.47 (7.94, 37-81) | 55.59 (7.87, 38-81) |
| PTSD symptom severity | 28.81 (13.40, 17-82) | 45.42 (15.00, 17-82) | 25.96 (10.78, 17-76) |
| Alcohol use | 3.25 (2.95, 0-21) | 3.59 (4.80, 0-19) | 3.23 (2.77, 0-21) |
| **N (%)** |  |  |  |
| Race (N, % white) | 881 (94.1%) | 115 (94.3%) | 634 (95.3%) |
| Ethnicity (N, % non-Hispanic) | 766 (94.2%) | 99 (92.5%) | 553 (94.4%) |
| Law enforcement | 603 (64.4%) | 68 (58.1%) | 440 (67.5%) |
| 9/11 dust cloud exposure | 178 (19.3%) | 28 (23.5%) | 125 (19.0%) |
| Lifetime PTSD diagnosis | 122 (15.5%) | 122 (100%) | 665 (100%) |
| Lifetime Depression diagnosis | 106 (13.0%) | 62 (53.9%) | 43 (6.6%) |

*Notes:*

Percentages were calculated based on available data (i.e. missing N was excluded from the denominator).

PTSD symptom severity was assessed using the PTSD Checklist (PCL)–Specific Version^1^; Alcohol use was assessed with the Alcohol Use Disorders Identification Test (AUDIT)^2^, Lifetime PTSD and Depression diagnoses were obtained using selected modules of the Structured Clinical Interview for DSM-IV^3^ or the Diagnostic Interview Schedule for DSM-IV^4^.

Cases and controls differed significantly on PTSD symptom severity (*t*(*df*)=16.87 (768), *p*<.001, *d*=11.62) and prevalence of lifetime depression diagnosis (*X*^2^(1, 766)=184.92, *p*<.001, *φ*=.49).

Supplementary Table 2 – Skew and kurtosis of PTSD symptom severity, multiprotein composite score, and 11 significant proteins.

|  | **Skew** | **Kurtosis** |
| --- | --- | --- |
| PTSD symptom severity | 1.34 | 1.10 |
| Multiprotein composite score | 0.40 | 0.32 |
| SKR3 | 0.72 | 2.53 |
| NCAN | -0.55 | 1.23 |
| BCAN | -0.06 | 0.12 |
| MSR1 | -0.01 | 0.70 |
| PVR | 0.10 | 0.62 |
| TNFRSF21 | 0.50 | 1.02 |
| DRAXIN | 0.58 | 1.32 |
| CLM6 | 0.29 | 0.65 |
| SCARB2 | 1.59 | 7.60 |
| CPM | -0.41 | 0.73 |
| SIGLEC1 | 0.07 | 1.16 |

Supplementary Figure 1 - Histograms of PTSD symptom severity, multiprotein composite score, and 11 significant proteins.

|  |  |
| --- | --- |
| 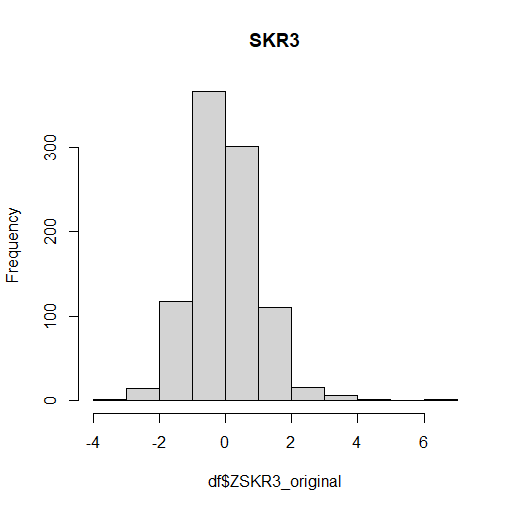 | 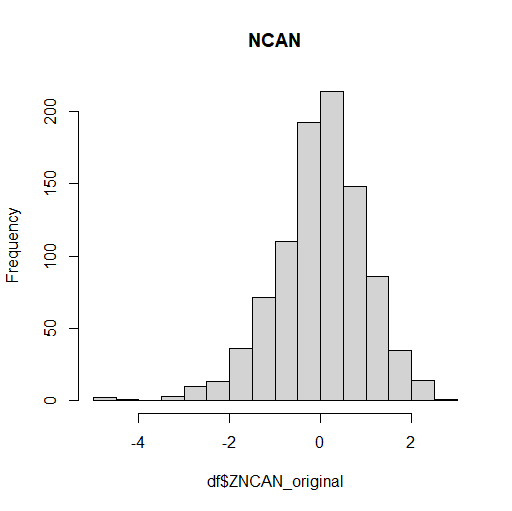 |
| 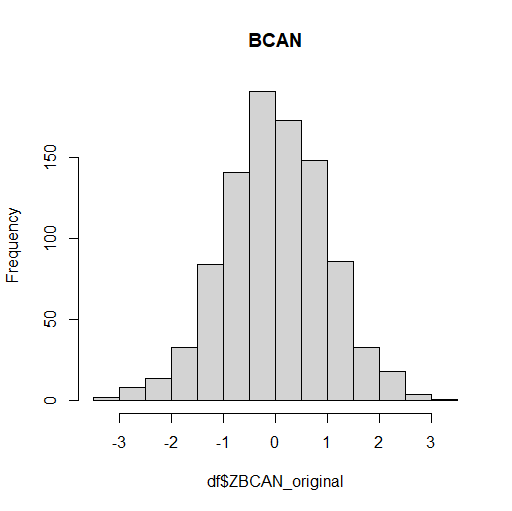 | 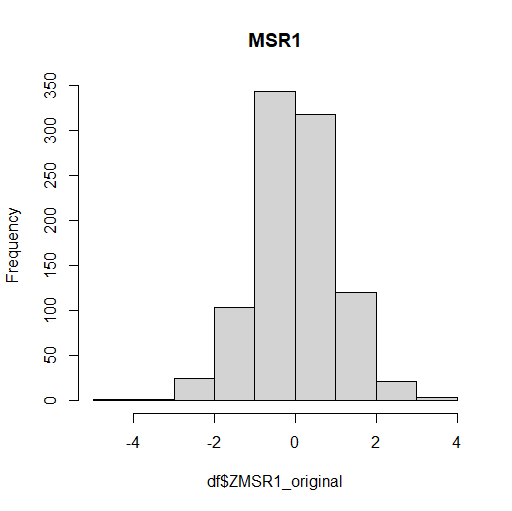 |
| 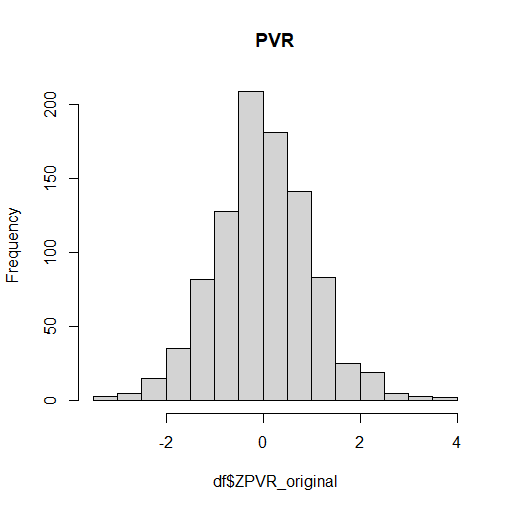 | 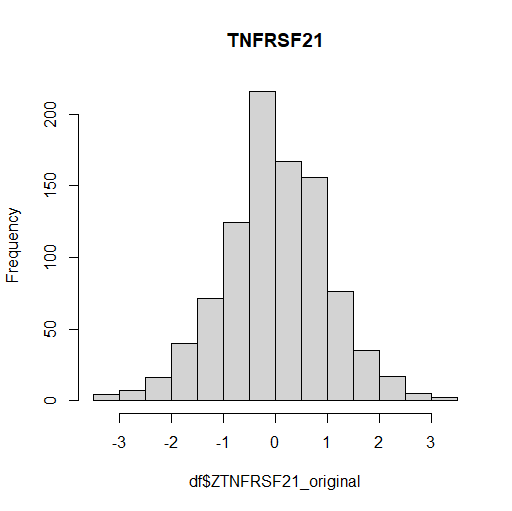 |
| 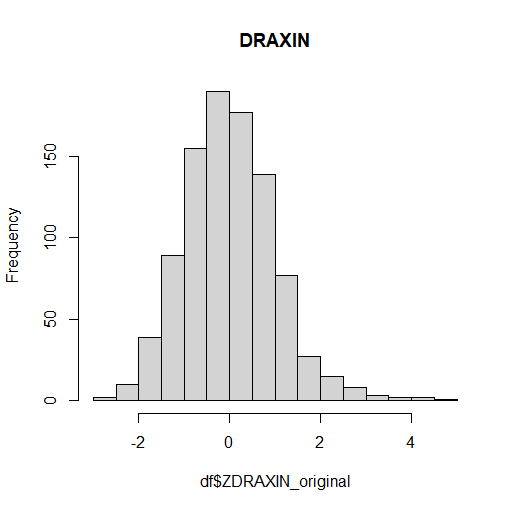 | 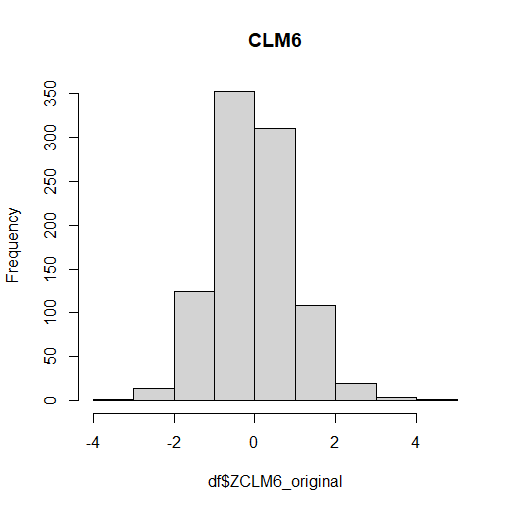 |
| 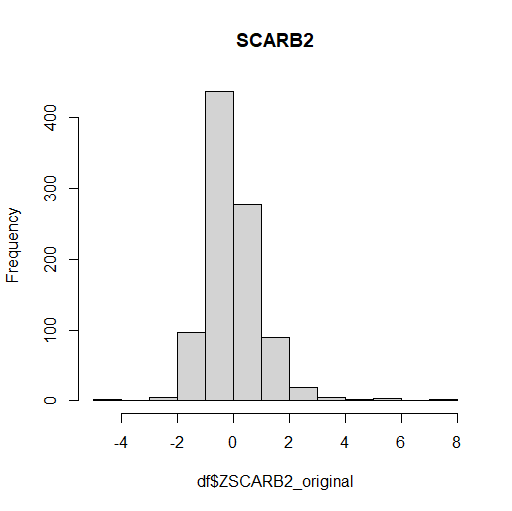 | 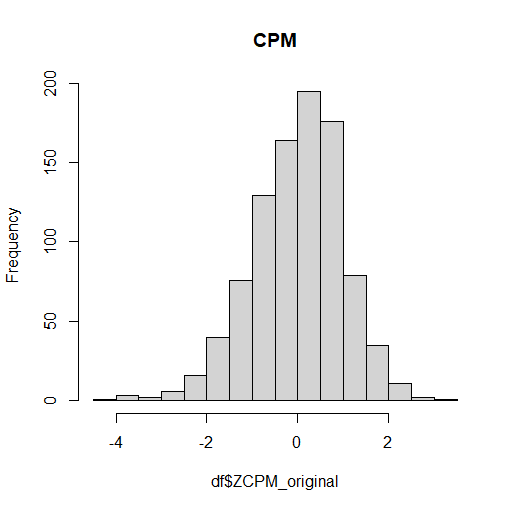 |
| 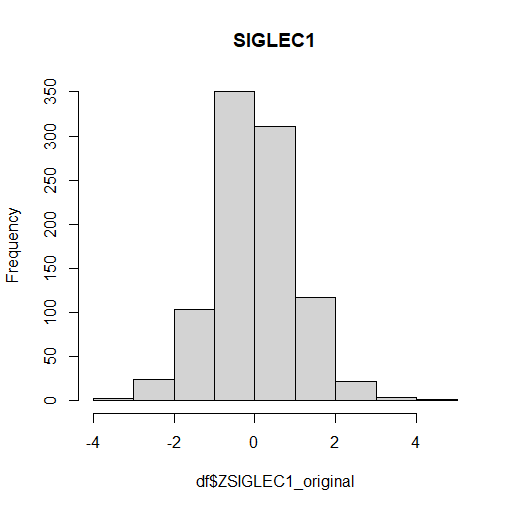 |  |

**Supplementary References**

1. The PTSD Checklist (PCL): Reliability, validity, and diagnostic utility. *Proceedings of the Annual Convention of the International Society for Traumatic Stress Studies*1993. International Society for Traumatic Stress Studies San Antonio.

2. Bohn MJ, Babor TF, Kranzler HR. The Alcohol Use Disorders Identification Test (AUDIT): validation of a screening instrument for use in medical settings. *Journal of studies on alcohol* 1995; **56**(4)**:** 423-432.

3. First MB, Spitzer RL, Gibbon M, Williams JB. *Structured Clinical Interview for DSM-IV Axis I Disorders: Patient Edition (February 1996 Final), SCID-I/P*. Biometrics Research Department, New York State Psychiatric Institute1998.

4. Robins L, Cottler L, Bucholz K, Compton W, North C, Rourke K. Diagnostic interview schedule for DSM-IV. St Louis, MO: Washington University Press1995.
